# Effects of Pre-Pregnancy Overweight/Obesity on the Pattern of Association of Hypertension Susceptibility Genes with Preeclampsia

**DOI:** 10.3390/life12122018

**Published:** 2022-12-03

**Authors:** Maria Abramova, Maria Churnosova, Olesya Efremova, Inna Aristova, Evgeny Reshetnikov, Alexey Polonikov, Mikhail Churnosov, Irina Ponomarenko

**Affiliations:** 1 Department of Medical Biological Disciplines, Belgorod State National Research University, 308015 Belgorod, Russia; 2 Department of Medical Genetics, Kharkiv National Medical University, 61022 Kharkov, Ukraine; 3 Grishchenko Clinic of Reproductive Medicine, 61052 Kharkov, Ukraine; 4 Department of Biology, Medical Genetics and Ecology and Research Institute for Genetic and Molecular Epidemiology, Kursk State Medical University, 305041 Kursk, Russia

**Keywords:** BMI, hypertension genes, preeclampsia, SNP, association

## Abstract

The aim of this study was to explore the effects of pre-pregnancy overweight/obesity on the pattern of association of hypertension susceptibility genes with preeclampsia (PE). Ten single-nucleotide polymorphisms (SNPs) of the 10 genome-wide association studies (GWAS)-significant hypertension/blood pressure (BP) candidate genes were genotyped in 950 pregnant women divided into two cohorts according to their pre-pregnancy body mass index (preBMI): preBMI ≥ 25 (162 with PE and 159 control) and preBMI < 25 (290 with PE and 339 control). The PLINK software package was utilized to study the association (analyzed four genetic models using logistic regression). The functionality of PE-correlated loci was analyzed by performing an in silico database analysis. Two SNP hypertension/BP genes, rs805303 *BAG6* (OR: 0.36–0.66) and rs167479 *RGL3* (OR: 1.86), in subjects with preBMI ≥ 25 were associated with PE. No association between the studied SNPs and PE in the preBMI < 25 group was determined. Further analysis showed that two PE-associated SNPs are functional (have weighty eQTL, sQTL, regulatory, and missense values) and could be potentially implicated in PE development. In conclusion, this study was the first to discover the modifying influence of overweight/obesity on the pattern of association of GWAS-significant hypertension/BP susceptibility genes with PE: these genes are linked with PE in preBMI ≥ 25 pregnant women and are not PE-involved in the preBMI < 25 group.

## 1. Introduction

Preeclampsia (PE) is a multisystem disorder of pregnancy previously defined by the onset of hypertension accompanied by significant proteinuria after 20 weeks of gestation [1]. PE is a major cause of maternal and perinatal mortality and morbidity associated with a number of complications for both mother and fetus [1,2]. PE affects 5% to 7% of all pregnant women and is responsible for over 70,000 maternal deaths and 500,000 fetal deaths worldwide every year [2]. PE is associated with a number of short- (intrauterine fetal death, preterm birth, fetal growth restriction, low Apgar score, etc.) and long-term (cerebral palsy, hearing loss, visual impairment, insulin resistance, etc.) perinatal and postnatal complications, including death [1]. However, despite its prevalence, well-cataloged risk factors, and clinical characteristics, the exact pathophysiology of this disorder remains yet unknown [2]. This knowledge deficit has hampered the development of targeted therapies and limited treatment options for healthcare providers [3].

The epidemiology of PE reflects a broad range of risk factors as well as the heterogeneity and complexity of the disease [1,4]. The maternal pre-existing risk factors for PE include race, age, family history of PE, BMI, somatic disorders (hypertension, renal disease, diabetes, etc.), smoking, etc. [1,2,4,5,6,7]. Genetic factors account for more than half of the liability of preeclampsia, and maternal genes contribute more than fetal genes (these data were obtained as a result of the analysis of pregnancy outcomes from 244,564 sibling pairs of families) [8]. It is believed that high BMI is one of the main risk factors for PE [4,6,9,10,11]. High BMI increases the overall PE risk by approximately 1.7–3.4-fold, and the risk of PE progressively rises with increasing BMI, even within the normal range [5,12]. Being overweight may be a stronger risk factor for severe PE than for mild disease [5]. The exact mechanisms linking overweight/obesity and PE remain unclear [1].

High BMI is a risk factor for both preeclampsia and cardiovascular disease [6]. Furthermore, preeclampsia is associated with an increased risk of later life cardiovascular disease [13]. These disorders share many pathophysiological features including oxidative stress, endothelial dysfunction, and increased inflammatory activation [6]. It should be emphasized that information on the genetic mechanisms by which obesity increases the risk of preeclampsia is limited [14]. It is important to note that only one work, a large-scale genetics-based analysis conducted by Venkatesh S. et al. in 2022, demonstrated causal association between overweight/obesity and PE: BMI was observationally (OR = 1.25) and genetically (OR = 2.09) associated with PE, and leptin, fasting insulin, and insulin resistance (each) mediated near 20—50% of the total genetically predicted association of obesity with PE [14].

Despite the numerous data pointing to (1) a strong association between pre-pregnancy BMI and the development of PE [5,6,9,10,11,12,14,15], (2) positive correlations of BMI with cardiovascular disease (hypertension, etc.) [16,17], (3) the impact role of the cardiovascular risk factors on PE [7,18], and (4) the significant role of genetic factors (including candidate genes of blood pressure, body weight, etc.) in the susceptibility to PE, etc. [8,19,20,21,22,23,24,25], genetic studies revealing the role of specific GWAS candidate genes of the cardiovascular disease in the formation of PE when interacting with BMI are extremely limited [14,26,27,28]. It has been hypothesized that pre-pregnancy overweight/obesity might influence the association between GWAS gene polymorphisms of hypertension and PE.

This study aimed to investigate the effects of pre-pregnancy overweight/obesity on the pattern of association of GWAS-significant hypertension susceptibility genes with PE.

## 2. Materials and Methods

### 2.1. Study Subjects

A case–control study was conducted and included 950 women divided into two groups as follows (according to their pre-pregnancy BMI): Group I—women with preBMI ≥ 25 (*n* = 321), included 162 women with PE and 159 control; Group II—women with preBMI < 25 (*n* = 629), included 290 women with PE and 339 control. PE was defined according to the recommendations of the American College of Obstetricians and Gynecologists (the presence of systolic and/or diastolic blood pressure ≥ 140 mm Hg and/or ≥ 90 mm Hg, respectively, and proteinuria with excretion of 0.3 g or more of protein in a 24 h urine specimen) [29]. The control groups (preBMI ≥ 25 and <25) consisted of women without PE. A clinical examination of all pregnant women was conducted by an experienced obstetrician at the perinatal center of the St. Joasaph Belgorod Regional Clinical Hospital. Inclusion criteria were the following: singleton pregnancy, 37–40 gestation weeks [23,30], and born in Central Russia and self-reported Russian origin [31]; exclusion criteria were the following: pregnant with pathological placental location, uterine leiomyoma, malformation disease of female reproductive organs, hepatic/renal failure, diabetes mellitus, and isosensitization of blood group systems (ABO/Rh factor) [32,33]. Written informed consent was obtained from all participating individuals, and the present study was approved by the Local Ethical Committee of the Belgorod State University.

The main exposure variable was preBMI based on maternal pre-pregnancy weight in kilograms and maternal height in centimeters from antenatal care visits. PreBMI was calculated as body weight in kilograms divided by height in meters squared (kg/m^2^) [34]. PreBMI was categorized according to WHO definitions as underweight < 18.5, normal weight 18.5–24.9, overweight 25.0–29.9, and obese ≥ 30.

### 2.2. DNA Isolation, Selection, and Genotyping SNPs

Genomic DNA was obtained from peripheral blood leucocytes by phenol/chloroform extraction, as previously described [35]. Ten common SNPs of hypertension susceptibility genes such as *AC026703.1* (rs1173771), *HFE* (rs1799945), *BAG6* (rs805303), *PLCE1* (rs932764), *OBFC1* (rs4387287), *ARHGAP42* (rs633185), *CERS5* (rs7302981), *ATP2B1* (rs2681472), *TBX2* (rs8068318), and *RGL3* (rs167479) were selected for the study based on their previously reported GWAS associations with hypertension/BP in Caucasian populations (Supplementary Table S1) and known functional relevance (Supplementary Table S2) [36,37]. The functionality of the selected loci was assessed by an in silico method using HaploReg online tools [38]: SNPs’ localization in evolutionarily conserved (measures evolutionary conservation GERP and SiPhy Cons) and DNase hypersensitive (“open” DNA) regions, correlation with chromatin structure (enhancer and promoter histone markers), SNP effects on regulatory motifs/proteins, and gene expression were examined. For all the samples, SNP genotyping was conducted by a Real-Time PCR System (CFX96, Real-Time PCR System (Bio-Rad Laboratories, Hercules, CA, USA)) [39,40] using necessary reagent kits (synthesized by TestGen, Ulyanovsk, Russia). During the experimental studies, quality control of genotyping was used. Firstly, all laboratory personnel were blinded to case–control status. Secondly, randomly selected samples (≈5%) were identified for duplicate testing [41]. Among these samples, the level of agreement with blinded duplicates was compiled 100%.

### 2.3. Statistical Analysis

Deviations from Hardy–Weinberg equilibrium (HWE) were assessed by the goodness-of-fit χ^2^ test [42]. The SNPs’ data were analyzed by logistic regression under dominant, additive, recessive, and allelic inheritance models [43] separately in two studied groups (preBMI ≥ 25 and <25). Baseline and clinical characteristics that could potentially influence the risk of PE separately in each study group of pregnant women (age, BMI, number of gravidity, spontaneous and induced abortions, presence of obesity, family history of PE for preBMI ≥ 25 group, and presence of family history of PE and tobacco consumption for preBMI < 25 group (Table 1)) were used as confounding factors in logistic regression models. To adjust for the multiple comparisons, an adaptive permutation testing was used [44,45]. The association analysis was performed using the PLINK package [46]. A p_perm_ level less than or equal to 0.025 was regarded as significant (a Bonferroni correction according to the number of the groups compared, *n* = 2, was additionally performed).

### 2.4. In Silico Bioinformatics Analysis of Functional SNPs

Computational predictions of functional impacts of PE susceptibility SNPs were considered by five in silico tools [47,48,49,50]: (i) SIFT [51], (ii) PolyPhen-2 [52], (iii) HaploReg [38], (iv) GTExproject [53], (v) Blood eQTL browser [54], (vi) GeneMANIA [55], (vii) Gene Ontology [56], (viii) STRING [57].

## 3. Results

Baseline and clinical data of preeclamptic and normotensive pregnant women with preBMI ≥ 25 (162 case and 159 control) and women with preBMI < 25 (290 cases and 339 control) are presented in Table 1. As shown in Table 1, the women with preBMI ≥ 25, PE cases vs. non-PE, were more likely to be older (*p* = 0.001) and had higher pre-pregnancy BMI (*p* = 0.0001), obesity (*p* = 0.0001), family history of PE (*p* = 0.02), mean number of gravidity (*p* = 0.03), and spontaneous (*p* = 0.008) and induced (*p* = 0.003) abortions. Among the women with preBMI < 25, PE cases vs. non-PE, there was a higher percentage of family history of PE (*p* = 0.0008) and lower proportion of tobacco consumption (*p* = 0.05). According to the received data, we used the abovementioned “specific” parameters for each study group of pregnant women (preBMI ≥ 25 and preBMI < 25) as covariates in the association regression analysis.

Among women with preBMI < 25 (Supplementary Table S3) and preBMI ≥ 25 (Supplementary Table S4), genotype frequencies were in Hardy–Weinberg equilibrium in the PE and control groups for all considered loci (after the Bonferroni correction for 10 SNPs P_bonf_ > 0.005).

The data association analysis in Table 2 showed that there were significant differences in the relationship between the genetic polymorphisms of the hypertension/BP genes and the PE in different preBMI groups. After adjustment for multiple comparisons (used Bonferroni correction), statistically significant associations (p_perm_ ≤ 0.025) between studied SNPs and risk of PE were found only among women with preBMI ≥ 25 (Table 2). In this cohort of subjects, two SNPs of hypertension/BP genes (rs805303 of the *BAG6* and rs167479 of the *RGL3*) were associated with PE. The polymorphic variant of SNP rs805303 (A allele) was negatively associated with PE using three genetic models: allelic (OR: 0.66, 95% CI: 0.48–0.92, *p*: 0.014, p_perm_: 0.019), additive (OR: 0.68, 95% CI: 0.49–0.93, *p*: 0.018, p_perm_: 0.020) and recessive (OR: 0.36, 95% CI: 0.18–0.74, *p*: 0.005, p_perm_: 0.006). The A allele of *BAG6* rs805303 was more frequent (1.31 times) in non-PE (0.386) compared to PE (0.294) participants (Supplementary Table S4). Along with this, the alternative variant of SNP rs167479 (G allele) was positively associated with PE (dominant model, OR: 1.86, 95% CI: 1.11–3.11, *p*: 0.019, p_perm_: 0.020). There was a prevalence (1.16 times) of allele G of *RGL3* rs167479 in PE subjects (0.522) compared to non-PE (0.449) (Supplementary Table S4).

There were no significant differences in the genotype/allele frequencies of the hypertension/BP gene polymorphism between preeclamptic patients and normotensive pregnant women with preBMI < 25 (p_perm_ > 0.025) (Table 2).

### In Silico Data of Functional PE-Associated SNPs

*SNP rs1674769*. Genetic variation caused by PE-associated SNP rs1674769, occurring in protein coding regions, alters the encoded amino acid at the mutated site and causes structural (Pro162His RGL3) and functional (predictive value is «deleterious» by SIFT and «probably damaging» by PolyPhen-2) changes in the mutated protein.

Using HaploReg v4.1, rs1674769 was predicted to localize in evolutionarily conserved regions (measures evolutionary conservation GERP and SiPhy Cons). DNase hypersensitivity—“open” DNA (Primary B cells from peripheral blood), enhancer (H1-derived trophoblast and mesenchymal stem cultured cells and placenta amnion) and promoter (placenta amnion and H1-derived neuronal progenitor and mesenchymal stem cells) histone markers, and nine motifs changed (AP-1, CCNT2, Rad21, SETDB1, SP1, TR4, WT1, ZNF219, and Zic). Importantly, the PE risk allele variant G of *RGL3* rs167479 causes an increase in affinity of the DNA TF-binding domains to six TFs—AP-1 (change in log-odds (LOD) score of G and T alleles: (2.2), CCNT2 (11.9), Rad21 (11.3), TR4 (4.3), WT1 (7.9), and ZNF219 (12.0)—and decreases affinity to three TFs—SETDB1 (−2.1), SP1 (−4.8), and Zic (−2.0). SNP rs1674769 has the most pronounced influence on affinity to CCNT2, Rad21, and ZNF219.

**Figure 1:**
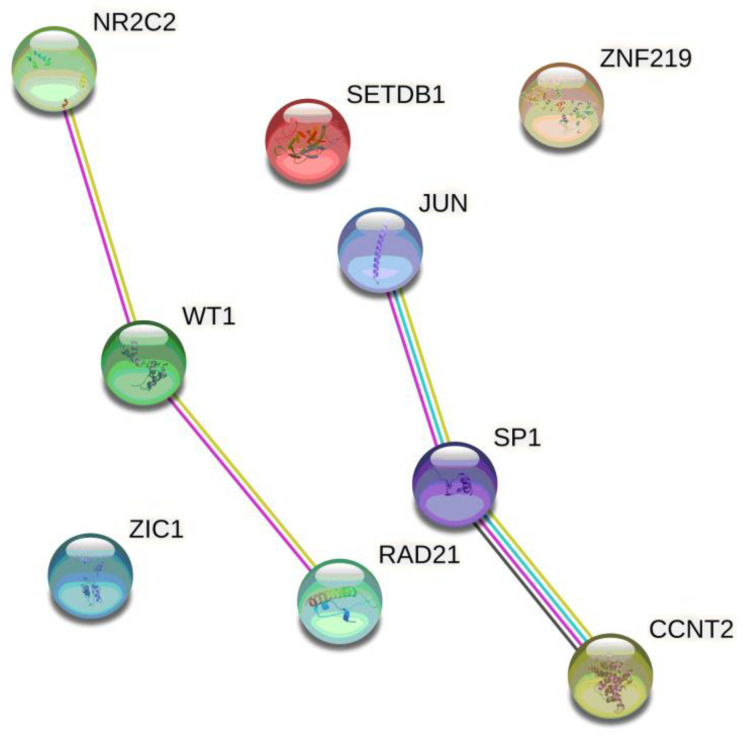
Interactomic networks involving transcription factors whose binding sites were in silico predicted at the PE risk allele at SNP rs167479 of *RGL3*. Note: JUN (AP-1)—Jun proto-oncogene, activator protein 1 transcription factor subunit; CCNT2—Cyclin-T2; RAD21—protein involved in DNA double-strand break repair; SETDB1—SET domain bifurcated histone lysine methyltransferase 1; SP1—specificity protein 1; NR2C2 (TR4)—nuclear receptor subfamily 2 group C member 2; WT1—Wilms tumor protein; ZNF219—zinc finger protein 219; ZIC1—zinc finger protein ZIC1.

Based on STRING resource annotations, the interactomic networks of nine TFs associated with the PE risk allele at SNP rs167479 of *RGL3* were visualized (Figure 1), and their biological pathways were identified. TFs predicted to bind with rs167479 were enriched with biological process/molecular function ontologies such as regulation of transcription by RNA polymerase II (GO: 0006357, FDR = 0.003), regulation of transcription, DNA-templates (GO: 0006355, FDR = 0.003), RNA polymerase II cis-regulatory region sequence-specific DNA binding (GO: 0000978, FDR = 0.004), DNA-binding transcription factor activity, RNA polymerase II-specific activity (GO: 0000981, FDR = 0.004), positive regulation of macromolecule metabolic process (GO: 0010604, FDR = 0.008), HMG box domain binding (GO: 0071837, FDR = 0.009), positive regulation of cellular process (GO: 0048522, FDR = 0.009), positive regulation by host of viral transcription (GO: 0043923, FDR = 0.019), response to hormone stimulus (GO: 00328704, FDR = 0.045), etc. According to the WikiPathways tools implemented in the STRING online resource, nine TFs that correlated with rs167479 were involved in the following pathways important for PE biology: androgen receptor signaling pathway (WP138, FDR = 0.014), estrogen receptor pathway (WP2881, FDR = 0.017), and estrogen signaling pathway (WP712, FDR = 0.033).

Using the public GTEx database, the expression quantitative effect of rs1674769 was observed: individuals carrying the PE risk G allele showed lower levels of expression of CTC-510F12 in the pituitary (Supplementary Table S5).

*SNP rs805303*. The effects of SNP rs805303 on chromatin structure and allele-specific transcription factor binding were identified using HaploReg. The intronic annotation of rs805303 *BAG6* indicated that it affected DNA motifs such as CACD (PE protective allele A decreases affinity to HIM, ΔLOD: −2.2) and that there was a direct effect on enhancer (hESC-derived CD56+ mesoderm cultured cells, H9-derived neuronal progenitor cultured cells, and Primary B and T cells (regulatory, effector/memory enriched, helper, etc.) from peripheral blood and brain (hippocampus middle, anterior caudate, dorsolateral and prefrontal cortex, etc., and male fetal brain, fetal adrenal gland, fetal muscle trunk, etc.)) and promoter (brain germinal matrix, adipose-derived mesenchymal stem cell cultured cells, adipose nuclei, etc.) histone markers.

In the Blood eQTL database, the minor allele of the genetic variant rs805303 was associated with decreased whole-blood mRNA expression of four genes: *LY6G5C* (Z-score: −13.34 *p* = 1.32 × 10^−40^), *HSPA1B* (Z-score: −12.75 *p* = 3.26 × 10^−37^), *HCP5* (Z-score: −4.25 *p* = 2.11 × 10^−5^), and *AIF1* (Z-score: −3.95 *p* = 7.82 × 10^−5^).

Based on in silico data of the GTEx tool, the pronounced tissue-specific gene expression effects of rs805303 were found (Supplementary Table S5). It was inferred from GTEx that rs805303 is associated with the expression of 35 genes in 44 tissues/organs (Supplementary Table S5). This locus regulates the expression level of many genes in the PE pathophysiology important organs (tissues), such as the brain (cortex, substantia nigra, basal ganglia, putamen, hypothalamus, pituitary, hippocampus, etc.) (*ABHD16A*, *CYP21A1P*, *DDAH2*, *LY6G5B*, *LY6G5C*, *LY6G6C*, and *MPIG6B*), whole blood (*LY6G5C*, *LY6G5B*, *HLA-DRB5*, *C4B*, *PRRC2A*, *CYP21A2*, *VWA7*, *C4A*, *CYP21A1P*, *AIF1*, and *C6orf48*), ovary (*LY6G5B*), thyroid (*LY6G5C*, *LY6G5B*, *HLA-DRB5*, *CCHCR1*, *CYP21A1P*, *C4A*, *RNF5*, *ABHD16A*, and *LY6G6F*), adrenal gland (*DDAH2*, *LY6G5B*, and *ABHD16A*), adipose (visceral and subcutaneous) (*ABHD16A*, *C4A*, *C6orf48*, *CCHCR1*, *CYP21A1P*, *CYP21A2*, *DDAH2*, *HLA-B*, *HLA-DRB5*, *LY6G5B*, *LY6G5C*, *STK19B*, and *VWA7*), etc. It is important to note that as a rule, individuals carrying the PE protective allele A rs805303 had lower gene expression (Supplementary Table S5).

The GTEx dataset demonstrated the regulatory role of rs805303 on the splicing patterns of mRNA precursors (Supplementary Table S6). The SNP rs805303 is associated with the alternative splicing traits (sQTL) of 17 genes in 38 tissues/organs (Supplementary Table S6). Tissue-specific genotype-splicing associations of rs805303 were registered in organs (tissues) involved in PE molecular pathways: the uterus (*BAG6*), multiple brain regions (*BAG6* and *LY6G5C*), visceral and subcutaneous adipose (*BAG6*, *HLA-DRB1*, *HLA-DRB5*, *HLA-DRB6*, and *AIF1*), the adrenal gland (*BAG6*, *CCHCR1*, *CYP21A1P*, and *CYP21A2*), the ovary (*BAG6*), the thyroid (*HLA-DRB1*, *HLA-DRB5*, *HLA-DRB6*, *GPANK1*, *STK19*, *STK19B*, *CCHCR1*, *BAG6*, and *FLOT1*), whole blood (*BAG6*, *AIF1*, *HLA-DRB1*, *HLA-DRB5*, *HLA-DRB6*, *GPANK1*, *GPANK1*, and *LY6G5C*), etc. (Supplementary Table S6). It should be noted that the sQTL effects of the rs805303 polymorphism in different organs (tissues) are multidirectional (positive/negative regulation splicing quantitative traits).

**Figure 2:**
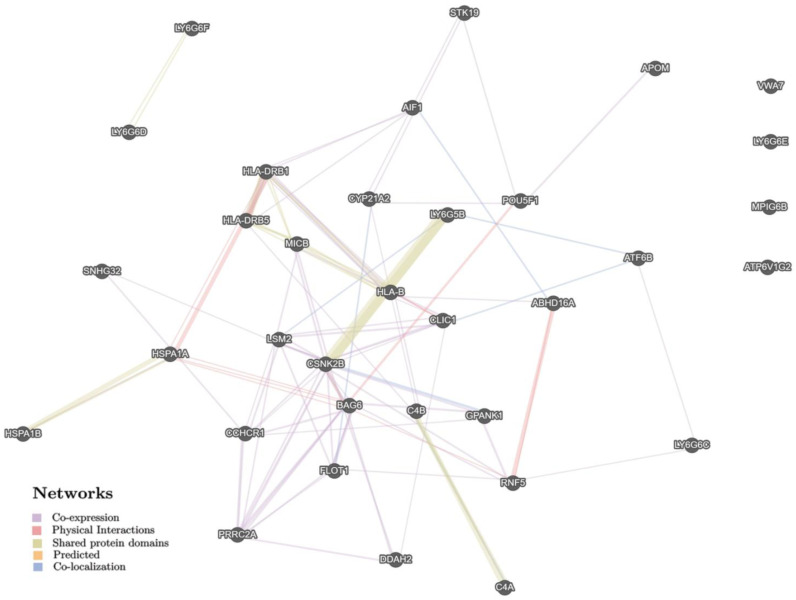
The interaction networks of the candidate genes associated with rs805303 (eQTL/sQTL/regulatory effects this SNP) inferred using GeneMANIA (http://genemania.org (accessed on 16 June 2022)).

**Figure 3:**
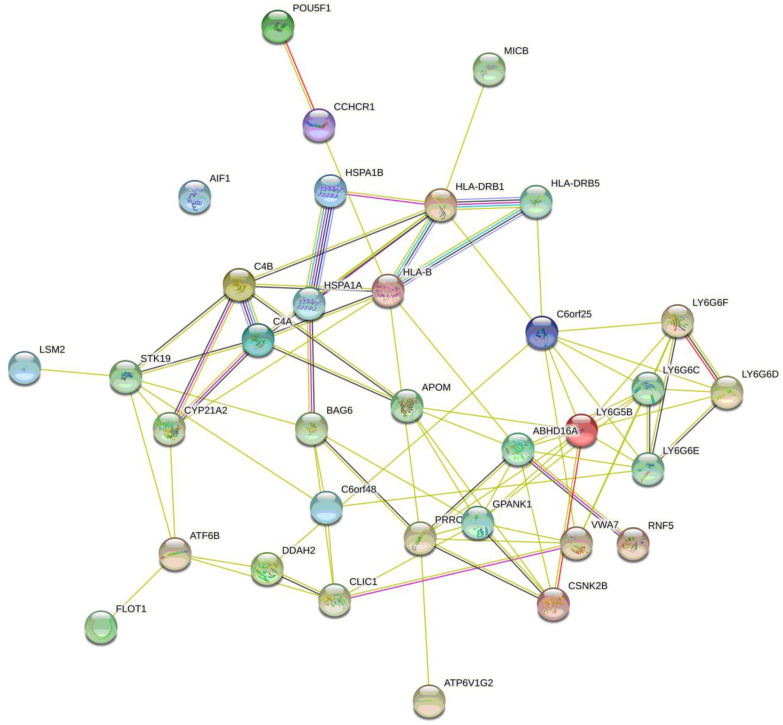
The protein–protein interaction networks of the candidate genes associated with rs805303 (eQTL/sQTL/regulatory effects this SNP) inferred using STRING (https://string-db.org/ (accessed on 16 June 2022)).

In summary, by in silico analysis, rs805303 was found to correlate with eQTL/sQTL/regulatory effects of 41 genes (Supplementary Table S2, Supplementary Table S5, and Supplementary Table S6). The gene–gene and protein–protein interactions of this set of genes are displayed in Figure 2 (defined by GeneMANIA) and Figure 3 (identified by STRING), respectively. The gene–gene interactions were performed through co-expression (64.82%), physical interaction (14.09%), shared protein domains (13.76%), co-localization (7.23%), and predicted interaction (0.09%) (Figure 2). The protein–protein interactions were carried out mainly due to protein clusters such as LY-6 antigen/upa receptor-like, acetylcholine receptor regulator activity (STK19, VWA7, LY6G6C, LY6G6D, LY6G6F, LY6G5B, GPANK1, PRRC2A, LY6G5C, LY6G6E, and ABHD16A) (FDR = 8.86 × 10^−16^), lymphocyte antigen 6G6E, lymphocyte antigen 6 complex locus protein G6D/G6F (LY6G6C, LY6G6D, LY6G6F, LY6G5B, LY6G5C, and LY6G6E) (FDR = 8.92 × 10^−10^), lymphocyte antigen 6 complex locus protein G6C, lymphocyte antigen 6 complex locus protein G5C (LY6G6C, LY6G6D, LY6G5B, and LY6G5C) (FDR = 6.06 × 10^−7^), proline-rich protein 3, trim10, ring-hc finger (VWA7, GPANK1, PRRC2A, and ABHD16A) (FDR = 9.06 × 10^−6^), phosphatidylserine lipase ABHD16A, and protein of unknown function DUF4661 (GPANK1, PRRC2A, ABHD16A) (FDR = 0.00012). The Gene Ontology enrichment on this set of genes/proteins (conducted by STRING) is blood microparticle (GO: 0072562, FDR = 0.005), extracellular exosome (GO: 0070062, FDR = 0.013), MHC protein complex (GO: 0042611, FDR = 0.013), integral component of lumenal side of endoplasmic reticulum (GO: 0071556, FDR = 0.013), ER to Golgi transport vesicle membrane (GO: 0012507, FDR = 0.033), positive regulation of immune system process (GO: 0002684, FDR = 0.045), cellular response to topologically incorrect protein (GO: 0035967, FDR = 0.045), response to organic substance (GO: 0010033, FDR = 0.045), and detection of external biotic stimulus (GO: 0098581, FDR = 0.048).

## 4. Discussion

This study was the first to identify a BMI-specific association of GWAS-significant hypertension/BP susceptibility genes with PE: rs805303 of *BAG6* (protective allele: A, OR: 0.36–0.66) and rs167479 of *RGL3* (risk allele: G, OR: 1.86) were associated with PE in preBMI ≥ 25 pregnant women and not associated with disorder in the preBMI < 25 group. Pronounced pleiotropic tissue-specific regulatory/expression/splicing effects of the PE-associated SNPs (rs1674769 and rs805303) were also documented. The most significant functionality (multi-expression and splicing patterns) was registered for rs805303, which affected 41 various genes.

Literature data overwhelmingly support that high BMI is a major risk factor for PE [4,6,10]. Overweight and obese women have, respectively, an increased risk of developing PE with severe features at ≥34 wks of gestation (overweight, OR = 1.4; obese, OR = 2.0) [15]. While numerous epidemiological studies have demonstrated that obesity/overweight increases the risk of PE, these mechanisms have yet to be fully elucidated [9]. It may be that placental, adipose tissue, and underlying endothelial dysfunction by metabolic factors such as leptin, fasting insulin, insulin resistance, etc., mediate the impact of obesity on increasing the risk for PE [10,11,58,59,60]. Spradley et al. [9] hypothesized that obesity-related metabolic factors increase the risk for developing PE by impacting various stages in the pathogenesis of PE (cytotrophoblast migration and placental ischemia; release of soluble placental factors into the maternal circulation; maternal endothelial and vascular dysfunction). Authors put forth the concept that obesity and metabolic factors such as lipids, insulin, glucose, and leptin affect placental function and increase the risk of developing hypertension in pregnancy by reducing placental perfusion, enhancing placental release of soluble factors, and by increasing the sensitivity of the maternal vasculature to placental ischemia-induced soluble factors [9]. BMI-related dyslipidemia and elevated C-reactive protein increase the risk of PE [61,62]. It should be noted that BMI may modify the association of C-reactive protein with PE [61].

Mendelian randomization has previously indicated causal associations of genetically predicted BMI and visceral adipose tissue (VAT) mass with PE (OR = 2.09 per 1 SD increase in obesity trait and OR = 3.08 per 1 kg increase in predicted VAT mass, respectively) (analyzed data of 257,193 women of European ancestry in UK Biobank and publicly available genome-wide association studies) [14]. Furthermore, the work [14] showed that on the one hand, leptin and insulin influence the risk of PE independently of obesity, but on the other hand, leptin, fasting insulin, and insulin resistance each mediated between 20% and 50% of the total genetically predicted association of obesity with PE.

Obesity/overweight and PE are diseases that result from multiple genetic and environmental factors [9]. These two conditions share many pathophysiological mechanisms, however, only 10% of obese women will develop PE [6,63]. Important here may be “additional” risk factors, including genetic ones, which increase the probability of developing PE for obese women. These genetic risk factor developments of PE for obese/overweight women can be GWAS-significant hypertension/BP susceptibility genes (*BAG6* and *RGL3*), which, according to the results of our work, are involved in the PE development in preBMI ≥ 25 pregnant women and not associated with disorder in the preBMI < 25 group. These genes may be part of shared genetic components of etiological relationships of overweight/obesity with PE.

According to data from earlier GWAS, rs805303 of *BAG6* was associated with levels of both systolic and diastolic BP and hypertension [64,65], and rs167479 of *RGL3* was associated with BP (systolic, diastolic and pulse, and mean arterial pressure) and hypertension [65,66,67,68,69,70,71]. Moreover, the materials we obtained on the protective effect of allele A rs805303 of *BAG6* (OR: 0.36–0.66) and the risk role of allele G rs167479 of *RGL3* (OR: 1.86) for PE in preBMI ≥ 25 pregnant women are fully consistent with the effects of these alleles, established in the above GWAS: allele A rs805303 *BAG6* was associated with low BP and decreased risk of hypertension and allele G rs167479 *RGL3* was correlated with high BP and elevated risk of hypertension.

It should be noted that our data on significant correlations between genetic predisposition to hypertension/BP and PE in preBMI ≥ 25 pregnant women are consistent with the results of previous studies on this issue [26,27,28]. According to the multi-ethnic maternal PE GWAS data from Gray et al., the disorder-associated locus rs9478812 *PLEKHG1* [28] has previously been implicated in GWAS of BP and BMI [72,73]. In the GWAS, Steinthorsdottir et al. found five variants (rs259983 *ZNF831* and rs1421085 *FTO* were associated on genome-wide significance, *p*  ≤  4 × 10^−9^; rs16998073 *FGF5*, rs3184504 *SH2B3*, and rs419076 *MECOM* were associated on significance 4 × 10^−9^ < *p* < 5.6 × 10^−5^) associating with PE through the maternal genome [27]. All of them have previously been GWAS-associated with BP [74,75,76], and one SNP, rs1421085 *FTO*, has also been GWAS-associated with BMI [77]. In addition, Steinthorsdottir et al. showed a positive genetic correlation between systolic and diastolic BP, hypertension, and PE (r_g_ = 0.3–0.4) and uncovered an association of the polygenic risk score for hypertension with PE [27]. Previously published GWAS completed by Gray et al. demonstrated that risk alleles for elevated DBP and increased BMI were most strongly associated with PE risk [28].

The specific genetic mechanisms by which known clinical risk factors contribute to PE development, including hypertension and obesity/overweight, have not been fully elucidated [28]. Gray et al. express the following proposed genetic mechanisms for these relationships [28]. Firstly, in previously published hypertension GWAS, risk loci are enriched for regulatory elements affecting gene expression in vascular endothelial cells and are associated with end organ damage in the heart, cerebral vessels, carotid artery, etc. [78]. As PE is characterized by diffuse endothelial dysfunction [2,3,7], women with genetic predisposition to altered vascular endothelial cell function are likely to be at high risk of PE. Secondly, in a published obesity GWAS, identified risk loci are highly associated with brain regions important for appetite regulation, learning, emotion, memory, insulin utilization, energy/lipid metabolism, and adipogenesis [79]. These pathways mediated through BMI-related effects may directly or indirectly contribute to PE pathophysiology, and in the opinion of Gray et al., it is an important question for future investigations [28].

One of the parts of the possible answer to the question about the mechanisms of BMI-related effects and their correlation with genetic predisposition to hypertension/BP with PE may be our data on the pronounced functional effects on GWAS-significant SNPs of the hypertension/BP susceptibility genes associated with PE. Regarding the specific genetic locus tagged by the study-significant SNP, rs805303, this SNP lies within an intronic region of *BAG6* in a region predicted to have an “active” promoter (marked by H3K9ac_Pro modified histone) in adipose-related cultured cells (mesenchymal-stem-cell-derived adipocyte, adipose-derived mesenchymal stem cell, and adipose nuclei). This genetic variant demonstrated the regulatory role for mRNA expression/splicing patterns of 35/17 genes in more than 35 tissues/organs, including 10/5 genes in visceral and subcutaneous adipose (*BAG6*, *CYP21A1P*, *CYP21A2*, *HLA-DRB1*, *HLA-DRB5*, *HLA-DRB6*, etc.), which enrich the regulation of immune system processes and organelle membrane components. The locus rs167479 of *RGL3* is situated in the enhancer and promoter in placenta amnion. This SNP causes structural (Pro162His) and functional («deleterious» predictive potential) changes in the RGL3 protein. The T to G change at rs167479 (G, effect allele; T, other allele) is predicted to alter the binding sites of nine transcription factors, AP-1, CCNT2, Rad21, SETDB1, SP1, TR4, WT1, ZNF219 and Zic, which are involved in the regulation of the gene transcription process and estrogen/androgen receptor signaling pathways. Thus, the regions tagged by rs167479 and rs805303 are likely to be functionally important and, consequently, may be the specific regions responsible for increasing/decreasing PE risk. It should be noted that it is interesting that one of the genes whose expression in nerve tissue is determined by rs805303 *POU5F1* (data obtained in silico in this work), together with genes *ESRRG* and *ZNF554* (due to gene transcription regulation), has the highest number of significant correlations with predominantly placenta-expressed genes, and, hence, may be deemed as a hub factor for PE development [80]. Than et al. pointed to the possible involvement of these genes in the dysregulation of trophoblast differentiation in preterm PE [80].

## 5. Conclusions

This study demonstrated a BMI-specific association of GWAS-significant hypertension/BP susceptibility genes with PE: a significant association was found between these genes and PE in preBMI ≥ 25 pregnant women, and their absence was found in the preBMI < 25 group. It was shown that genetic predisposition to hypertension/BP is an important risk factor for PE in overweight/obese women.

## Figures and Tables

**Table 1:** Phenotypic characteristics of the study participants.

Parameters	PreBMI ≥ 25			PreBMI < 25		
	PE Patients $\bar{X}\;\;\pm \;\mathrm{SD}/\%\;(n)$	Controls $\bar{X}\;\;\pm \;\mathrm{SD}/\%\;(n)$	*p*	PE Patients $\bar{X}\;\;\pm \;\mathrm{SD}/\%\;(n)$	Controls $\bar{X}\;\;\pm \;\mathrm{SD}/\%\;(n)$	*p*
*N*	162	159	**-**	290	339	-
Age, years(min–max)	29.05 ± 5.09 (18–42)	27.09 ± 5.31 (18–41)	**0.001**	26.48 ± 4.83 (17–43)	26.36 ± 4.77 (16–42)	0.71
Pre-pregnancy BMI, kg/m^2^	30.50 ± 4.51	27.82 ± 2.45	**0.0001**	21.71 ± 1.89	21.60 ± 2.02	0.39
Family history of preeclampsia	24.69 (40)	13.84 (22)	**0.02**	23.10 (67)	11.50 (39)	**0.0008**
Smoker (yes)	48.76 (79)	52.83 (84)	0.54	44.48 (129)	52.80 (179)	**0.05**
Alcohol consumption (yes)	77.78 (126)	83.65 (133)	0.23	74.14 (215)	77.29 (262)	0.41
Pre-pregnancy blood pressure (BP)						
Systolic BP, mm Hg	113.82 ± 9.65	113.71 ± 6.54	0.78	111.95 ± 9.99	110.59 ± 9.26	0.08
Diastolic BP, mm Hg	72.30 ± 6.44	72.82 ± 6.30	0.54	71.68± 5.36	71.02 ± 4.24	0.56
Mean BP, mm Hg	86.14 ± 7.30	86.62 ± 6.33	0.22	85.24 ± 8.16	84.88 ± 7.65	0.13
Pulse BP, mm Hg	40.32 ± 4.91	39.99 ± 3.74	0.11	39.67 ± 4.36	38.57 ± 4.24	0.09
Age at menarche and menstrual cycle						
Age at menarche, years	12.15 ± 1.17	12.39 ± 1.01	0.45	12.72 ± 0.93	12.67 ± 1.09	0.11
Duration of menstrual bleeding (mean, days)	4.85 ± 1.32	5.03 ± 0.76	0.16	4.93 ± 1.10	4.99 ± 0.75	0.48
Menstrual cycle length (mean, days)	27.41 ± 3.13	28.16 ± 1.16	0.13	28.14 ± 2.67	28.48 ± 1.87	0.59
Reproductive status						
First pregnancy	35.80 (58)	37.74 (60)	0.81	41.38 (120)	44.54 (151)	0.47
No. of gravidity (mean)	2.14 ± 1.88	1.41 ± 1.67	**0.03**	1.10 ± 1.33	1.01 ± 1.33	0.36
No. of births (mean)	0.81 ± 0.89	0.79 ± 0.87	0.76	0.40 ± 0.54	0.41 ± 0.63	0.62
No. of spontaneous abortions (mean)	0.28 ± 0.52	0.12 ± 0.35	**0.008**	0.22 ± 0.47	0.16 ± 0.42	0.08
No. of induced abortions (mean)	0.98 ± 1.33	0.48 ± 0.88	**0.003**	0.45 ± 0.76	0.43 ± 0.82	0.38
No. of stillbirths	0.07 ± 0.25	0.02 ± 0.14	0.08	0.03 ± 0.18	0.01 ± 0.13	0.07
Somatic pathologies						
Cardiovascular	17.90 (29)	10.69 (17)	0.09	11.38 (33)	9.14 (31)	0.42
Kidney	7.41 (12)	5.03 (8)	0.52	4.14 (12)	2.95 (10)	0.56
Endocrine	3.70 (6)	3.77 (6)	0.99	2.76 (8)	0.88 (3)	0.14
Gastrointestinal	3.09 (5)	1.26 (2)	0.46	1.72 (5)	3.54 (12)	0.25
Obesity	46.91 (76)	20.13 (32)	**0.0001**	-	-	-

Note: *p* values were calculated by comparing PE patients and controls in each studied female cohort (preBMI ≥ 25 and preBMI < 25); *p* values < 0.05 are shown in bold.

**Table 2:** Associations of the studied gene polymorphisms with preeclampsia among preBMI < 25 and preBMI ≥ 25 female.

Chr	SNP	Minor Allele	*n*	Allelic Model				Additive Model				Dominant Model				Recessive Model			
				OR	95%CI		*p*	OR	95%CI		*p*	OR	95%CI		*p*	OR	95%CI		p
					L95	U95			L95	U95			L95	U95			L95	U95	
female with preBMI < 25																			
5	rs1173771	A	609	0.83	0.66	1.05	0.113	0.83	0.65	1.04	0.107	0.78	0.55	1.10	0.154	0.78	0.51	1.18	0.241
6	rs1799945	G	624	0.97	0.74	1.28	0.842	0.97	0.73	1.29	0.837	0.89	0.64	1.23	0.481	1.81	0.73	4.48	0.202
6	rs805303	A	628	0.86	0.69	1.08	0.199	0.85	0.67	1.08	0.187	0.83	0.60	1.15	0.260	0.79	0.50	1.26	0.321
10	rs932764	A	618	0.95	0.76	1.19	0.672	0.95	0.77	1.19	0.678	1.05	0.74	1.49	0.797	0.83	0.58	1.21	0.334
10	rs4387287	A	587	0.90	0.67	1.20	0.468	0.90	0.67	1.20	0.465	0.87	0.62	1.22	0.420	0.95	0.39	2.33	0.915
11	rs633185	G	620	1.02	0.80	1.30	0.891	1.02	0.79	1.32	0.885	1.07	0.78	1.46	0.696	0.87	0.45	1.66	0.665
12	rs7302981	A	621	1.06	0.84	1.33	0.644	1.06	0.84	1.34	0.635	0.99	0.71	1.37	0.943	1.28	0.80	2.03	0.302
12	rs2681472	G	625	1.16	0.84	1.60	0.360	1.16	0.84	1.61	0.356	1.13	0.79	1.61	0.500	2.06	0.60	7.11	0.253
17	rs8068318	C	603	1.18	0.92	1.52	0.198	1.18	0.92	1.51	0.201	1.11	0.80	1.53	0.528	1.74	0.96	3.15	0.068
19	rs167479	G	616	0.79	0.63	0.98	0.036	0.79	0.63	0.99	0.038	0.71	0.49	1.02	0.060	0.75	0.52	1.09	0.129
female with preBMI ≥ 25																			
5	rs1173771	A	315	0.98	0.71	1.34	0.880	0.97	0.71	1.35	0.877	0.92	0.57	1.49	0.741	1.04	0.58	1.84	0.905
6	rs1799945	G	319	1.15	0.77	1.70	0.494	1.14	0.78	1.67	0.507	1.00	0.63	1.59	0.998	2.82	0.88	9.06	0.081
6	rs805303	A	318	**0.66**	**0.48**	**0.92**	**0.014**	**0.68**	**0.49**	**0.93**	**0.018**	0.73	0.47	1.15	0.173	**0.36**	**0.18**	**0.74**	**0.005**
10	rs932764	A	318	1.02	0.74	1.39	0.927	1.01	0.74	1.38	0.927	1.22	0.75	1.98	0.418	0.82	0.48	1.40	0.460
10	rs4387287	A	306	0.89	0.59	1.36	0.592	0.89	0.58	1.36	0.588	0.81	0.50	1.31	0.390	1.69	0.40	7.20	0.478
11	rs633185	G	317	0.89	0.62	1.26	0.503	0.89	0.62	1.26	0.501	0.87	0.56	1.35	0.533	0.83	0.35	1.98	0.670
12	rs7302981	A	316	0.80	0.58	1.11	0.184	0.79	0.56	1.11	0.172	0.81	0.52	1.27	0.358	0.60	0.30	1.22	0.160
12	rs2681472	G	317	0.99	0.64	1.52	0.953	0.99	0.65	1.50	0.955	0.96	0.58	1.57	0.864	1.17	0.35	3.91	0.800
17	rs8068318	C	314	0.87	0.61	1.23	0.420	0.87	0.62	1.23	0.430	0.84	0.54	1.30	0.428	0.85	0.38	1.89	0.682
19	rs167479	G	317	1.34	0.98	1.83	0.067	1.36	0.99	1.88	0.062	**1.86**	**1.11**	**3.11**	**0.019**	1.19	0.70	2.02	0.520

Note: The indicators of the association of SNP with PE (OR, 95% CI, *p* values) were calculated by comparing PE patients and controls in each studied female cohort (preBMI ≥ 25 and preBMI < 25); all results were obtained after adjustment for covariates; OR: odds ratio; 95% CI: 95% confidence interval; *p* values < 0.05 are shown in bold.

## Data Availability

The data generated in the present study are available from the corresponding author upon reasonable request.

## Supplementary Materials

The following supporting information can be downloaded at: https://www.mdpi.com/article/10.3390/life12122018/s1, Table S1: The GWAS data regarding associations of the studied candidate gene polymorphisms with blood pressure/hypertension, some cardiovascular diseases, and anthropometric characteristics; Table S2: The regulatory potential of the studied SNPs; Table S3: The allele and genotype frequencies of the studied SNPs in the preeclampsia and control groups with preBMI < 25; Table S4: The allele and genotype frequencies of the studied SNPs in the preeclampsia and control groups with preBMI ≥ 25; Table S5: The eQTL effects of the two PE-associated SNPs (rs805303 and rs167479) in various tissues/organs; Table S6: The sQTL effects of the PE-associated polymorphism rs805303 in various tissues/organs. References [80,81,82,83,84,85,86,87,88,89,90,91,92,93] have cited in Supplementary Materials.
