# Supplementary material for: Effects of Pre-Pregnancy Overweight/Obesity on the Pattern of Association of Hypertension Susceptibility Genes with Preeclampsia
Source: Life (Basel). 2022 Dec 3;12(12):2018. doi: 10.3390/life12122018 (PMC9784908; doi:10.3390/life12122018)
Supplement: Supplementary file 1 [file life-12-02018-s001.zip › Suppl Table S1.pdf]

Supplementary table S1

The GWAS data about associations of the studied candidate genes polymorphisms with blood pressure/hypertension, some cardiovascular diseases and anthropometric characteristics

SNP of gene (position (hg38))	Phenotype	Association (significance) (affected allele)	Reference
rs1173771 *AC026703.1* (5: 32814922)	SBP	$\beta = 0.50$ ($p = 1.8\times10^{-16}$) (G)	International Consortium et al., 2011
	DBP	$\beta = 0.26$ ($p = 9.1\times10^{-12}$) (G)	
	Hypertension	$\beta = 0.06$ ($p = 3.2\times10^{-10}$) (G)	
	MAP	$\beta = 0.28$ ($p = 3.51\times10^{-9}$) (G)	Wain L. V., et al., 2011
	PP	$\beta = 0.28$ ($p = 4.56\times10^{-9}$) (G)	
	MAP	$\beta = -0.35$ ($p = 3.19\times10^{-8}$) (A)	Kato N., et al., 2015
	Hip circumference (sex-combined)	$\beta = 0.03$ ($p = 6.13\times10^{-13}$) (A)	Shungin D., et al., 2015
	Hip circumference (men)	$\beta = 0.03$ ($p = 6.19\times10^{-8}$) (A)	
	SBP	$\beta = -0.43$ ($p = 2.0\times10^{-28}$) (A)	Hoffmann T. J. et al., 2017
	PP	$\beta = -0.25$ ($p = 1.0\times10^{-21}$) (A)	
	DBP	$\beta = -0.19$ ($p = 8.0\times10^{-16}$) (A)	
	Height	$\beta = 0.04$ ($p = 9.0\times10^{-8}$) (A)	Tachmazidou I., et al., 2017
	SBP	$\beta = 0.51$($p = 3.20\times10^{-12}$) (G)	Wain L. V., et al., 2017
	PP	$\beta = 0.28$ ($p = 2.36\times10^{-9}$) (G)	
	SBP (smoking interaction) (European)	$p = 1.74\times10^{-38}$	Sung Y. J., et al., 2018
	DBP (smoking interaction) (European)	$p = 3.62\times10^{-22}$	
	SBP (smoking interaction) (trans-ethnic data)	$p = 8.25\times10^{-44}$	
	DBP (smoking interaction) (trans-ethnic data)	$p = 2.75\times10^{-28}$	
	SBP	$\beta = -0.42$ ($p = 4.19\times10^{-8}$) (A)	Takeuchi F., et al., 2018
	MAP	$\beta = -0.29$ ($p = 5.39\times10^{-8}$) (A)	
rs1799945 *HFE* (6: 26090951)	DBP	$\beta = 0.46$ ($p = 1.5\times10^{-15}$) (G)	International Consortium et al., 2011
	SBP	$\beta = 0.62$ ($p = 7.7\times10^{-12}$) (G)	
	Hypertension	$\beta = 0.10$ ($p = 1.8\times10^{-10}$) (G)	
	DBP	$\beta = -0.43$ ($p = 3.1 \times10^{-16}$) (C)	Ehret G. B. et al., 2016
	SBP	$\beta = -0.60$ ($p = 3.28 \times10^{-12}$) (C)	
	DBP	$\beta = -0.29$ ($p = 1.0\times10^{-18}$) (C)	Hoffmann T. J. et al., 2017

	SBP	β = −0.36 (p = 4.0×10^{-11}) (C)	
	DBP	β =0.52 (p = 9.2×10^{-18}) (G)	Liu C. et al., 2016
	MAP	β = 0.58 (p = 1.0×10^{-17}) (G)	
	SBP	β = 0.70 (p = 6.2×10^{-13}) (G)	
	Hypertension	Z score = 0.039 (p = 3.7×10^{-9}) (G)	
	DBP (trans-ethnic data)	β = −0.03 (p = 1.26×10^{-19}) (C)	Surendran P. et al., 2016
	DBP (European)	β = −0.03 (p = 9.82×10^{-10}) (C)	
	DBP	β = 0.47 (p = 8.87×10^{-14}) (G)	Wain L. V. et al., 2017
	SBP	β = 0.63 (p = 7.63×10^{-10}) (G)	
	DBP (smoking interaction) (European)	p = 1.51×10^{-31}	Sung Y. J. et al., 2018
	SBP (smoking interaction) (European)	p = 8.46×10^{-17}	
	DBP (smoking interaction) (trans-ethnic data)	p = 3.77×10^{-33}	
	SBP (smoking interaction) (trans-ethnic data)	p = 7.43×10^{-19}	
rs805303 *BAG6/ BAT2-BAT5* (6: 31648589)	SBP	β = 0.37 (p = 1.5×10^{-11}) (G)	International Consortium et al., 2011
	DBP	β = 0.23 (p = 3.0×10^{-11}) (G)	
	Hypertension	β = 0.05 (p = 1.1×10^{-10}) (G)	
	SBP (European)	β = −0.02 (p = 3.02×10^{-6}) (A)	Surendran P. et al., 2016
rs932764 *PLCE1* (10: 94136183)	SBP	β = 0.48 (p = 7.1×10^{-16}) (G)	International Consortium et al., 2011
	Hypertension	β = 0.06 (p = 9.4×10^{-9}) (G)	
	SBP	β = −0.50 (p = 6.88×10^{-17}) (A)	Ehret G. B., et al., 2016
	DBP	β = −0.22 (p = 6.28×10^{-10}) (A)	
	SBP	β = −0.30 (p = 1.0×10^{-14}) (A)	Hoffmann T. J. et al., 2016
	PP	β = −0.16 (p = 3.0×10^{-10}) (A)	
	PP	β = 0.26 (p = 5.67×10^{-8}) (G)	Wain L. V., et al., 2017
rs4387287 *OBFC1* (10:103918139)	DBP (European)	β= 0.22 (p=5.55 x 10^{-8}) (A)	Surendran P. et al., 2016
	SBP (European)	β= 0.34 (p=2.21 x 10^{-7}) (A)	
	DBP (trans-ethnic data)	β= 0.22 (p=4.21 x 10^{-10}) (A)	
	SBP (trans-ethnic data)	β= 0.36 (p=9.12 x 10^{-10}) (A)	
	Hypertension (trans-ethnic data)	Z score = 5.58 (p = 2.37 x 10^{-8}) (A)	
rs633185 *ARHGAP42*	SBP	β = −0.57(p = 1.2×10^{-17}) (G)	International Consortium et al., 2011
	DBP	β = −0.33(p = 2.0×10^{-15}) (G)	

(11:100722807)	Hypertension	$\beta = -0.07(p = 6.4\times10^{-11})$ (G)	
	SBP	$\beta = 0.52$ $(p = 6.97\times10^{-15})$ (C)	Ehret G. B., et al., 2016
	DBP	$\beta = 0.29$ $(p = 2.38\times10^{-12})$ (C)	
	SBP	$\beta = -0.49$ $(p = 1.0\times10^{-31})$ (G)	Hoffmann T. J. et al., 2016
	DBP	$\beta = -0.27$ $(p = 1.0\times10^{-26})$ (G)	
	PP	$\beta = -0.22$ $(p = 1.0\times10^{-15})$ (G)	
	SBP	$\beta = 0.52$ $(p = 8.43\times10^{-11})$ (C)	Wain L. V., et al., 2017
	DBP	$\beta = 0.27$ $(p = 2.33\times10^{-8})$ (C)	
	SBP x alcohol consumption interaction	$\beta = 0.54$ $(p = 2.24\times10^{-29})$ (C)	Feitosa M. F. et al., 2018
	MAP x alcohol consumption interaction	$\beta = 0.22$ $(p = 2\times10^{-12})$ (C)	
	MAP	$\beta = 0.39$ $(p = 2.56\times10^{-13})$ (C)	Takeuchi F., et al., 2018
	SBP	$\beta = 0.51$ $(p = 7.16\times10^{-12})$ (C)	
	DBP	$\beta = 0.33$ $(p = 8.93\times10^{-12})$ (C)	
	Hypertension	$\beta = 0.08$ $(p = 5.13\times10^{-10})$ (C)	
	Coronary artery disease	$(p = 8.81\times10^{-9})$ (C)	Zhou W., et al., 2018
	High blood pressure and chronic obstructive pulmonary disease	(p = 1.18×10^{-47})	Zhu Z., et al., 2019
	SBP (smoking interaction) (European)	$p = 8.44\times10^{-30}$	Sung Y. J., et al., 2018
	DBP (smoking interaction) (European)	$p = 2.68\times10^{-30}$	
	SBP (smoking interaction) (trans-ethnic data)	$p = 1.80\times10^{-40}$	
	DBP (smoking interaction) (trans-ethnic data)	$p = 1.18\times10^{-40}$	
	MAP	$\beta = 0.03$ $(p = 3.05\times10^{-50})$ (C)	Sakaue S. et al., 2021
rs7302981 *CERS5/AC074032.1* (12:50144032)	Hypertension	Z score = 6.23 ($p= 4.8 \times 10^{-10}$) (A)	Liu C. et al., 2016
	SBP	$\beta = 0.37$ $(p=9.4 \times 10^{-15})$ (A)	
	DBP	$\beta = 0.25$ ($p = 9.4 \times 10^{-19}$) (A)	
	DBP (European)	$\beta = 0.25$ ($p = 1.38 \times 10^{-17}$) (A)	Surendran P. et al., 2016
	SBP (European)	$\beta = 0.34$ ($p = 6.06 \times 10^{-13}$) (A)	
	Hypertension (European)	Z score = 6.07 $(p = 1.28 \times 10^{-9})$ (A)	
	DBP (trans-ethnic data)	$\beta = 0.25$ ($p = 2.60 \times 10^{-19}$) (A)	
	SBP (trans-ethnic data)	$\beta = 0.35$ ($p = 9.94 \times 10^{-19}$) (A)	
	Hypertension (trans-ethnic data)	Z score = 6.17 $(p = 6.82 \times 10^{-10})$ (A)	
rs2681472 *ATP2B1*	DBP	$\beta = 0.50$ $(p = 1.47\times10^{-9})$ (A)	Levy D. et al., 2009
	Hypertension	$\beta = 0.15$ $(p = 1.75\times10^{-11})$ (A)	

(12: 89615182)	Coronary artery disease	OR = 1.08 (p = 6.17×10^{-11}) (G)	Nikpay M., et al., 2015
	Myocardial infarction	OR = 1.08 (p = 6.03×10^{-9}) (G)	
	MAP	β = −0.59 (p = 1.1×10^{-21}) (G)	Liu C. et al., 2016
	SBP	β = −0.85 (p = 1.3×10^{-21}) (G)	
	DBP	β = −0.47 (p = 3.7×10^{-17}) (G)	
	Hypertension	β = −0.033 (p = 3.5×10^{-8}) (G)	
	Coronary artery disease	OR = 1.07 (p = 1×10^{-21}) (G)	Nelson C. P., et al., 2017
	SBP	β = 0.72 (p = 1.06×10^{-20}) (A)	Takeuchi F., et al., 2018
	DBP	β = 0.33 (p = 3.77×10^{-11}) (A)	
	MAP	β = 0.46 (p = 5.05×10^{-17}) (A)	
	PP	β = 0.40 (p = 5.49×10^{-14}) (A)	
	Hypertension	β = 0.07(p = 1.41×10^{-6}) (A)	
	Coronary artery disease (trans-ethnic data)	β = 0.060 (p= 6.8 x 10^{-25}) (G)	Koyama S., et al., 2020
	Coronary artery disease (Japanese)	β = 0.068 (p= 2.6 x 10^{-11}) (G)	
	Myocardial infarction	OR = 1.07 (p = 1.3×10^{-12}) (G)	Hartiala J. A., et al., 2020
	Myocardial infarction	β = 0.071 (p= 1.17 x 10^{-11}) (G)	Sakaue S. et al., 2021
rs8068318<br>*TBX2/*<br>*TBX2-AS1*<br>(17:61406405)	MAP	β = −0.28 (p = 2.0×10^{-8}) (C)	Liu C. et al., 2016
	SBP	β = −0.42 (p = 3.9×10^{-17}) (C)	
	Hypertension	Z score = −6.96 (p = 3.0 x 10^{-12}) (C)	
	DBP	β = −0.26 (p = 3.0×10^{-18}) (C)	
	SBP (European)	β = 0.42 (p= 1.3 x 10^{-15}) (T)	Surendran P. et al., 2016
	DBP (European)	β = 0.26 (p= 1.95 x 10^{-16}) (T)	
	Hypertension (European)	Z score = 6.97 (p = 3.21 x 10^{-12}) (T)	
	DBP (trans-ethnic data)	β = 0.25 (p= 2.75 x 10^{-18}) (T)	
	SBP (trans-ethnic data)	β = 0.41 (p= 2.3 x 10^{-17}) (T)	
	Hypertension (trans-ethnic data)	Z score = 6.96 (p = 3.43 x 10^{-12}) (T)	
rs167479<br>*RGL3*<br>(19:11416089)	SBP	β = 0.41 (p = 1.6 x 10^{-21}) (G)	Hoffmann T. J. et al., 2017
	DBP	β = 0.25 (p = 4.3 x 10^{-22}) (G)	
	PP	β = 0.18 (p = 3.2 x 10^{-8}) (G)	
	MAP	β = −0.30 (p = 7.3 x 10^{-11}) (T)	Liu C. et al., 2016
	Hypertension	Z score = −7.72 (p = 1.2 x 10^{-14}) (T)	
	SBP	β = −0.45 (p = 1.0 x 10^{-23}) (T)	
	DBP	β = −0.30 (p = 4.2×10^{-28}) (T)	
	DBP (European)	β= −0.33 (p = 1.99 x 10^{-31}) (T)	Surendran P. et al., 2016
	SBP (European)	β= −0.50 (p =1.49 x 10^{-26}) (T)	

	Hypertension (European)	Z score = −7.86 (p = 4.01 x 10^{-15}) (T)	
	DBP (trans-ethnic data)	β= −0.31 (p = 2.76 x 10^{-32}) (T)	
	SBP (trans-ethnic data)	β= −0.47 (p = 8.64 x 10^{-27}) (T)	
	Hypertension (trans-ethnic data)	Z score = −7.88 (p = 3.37 x 10^{-15}) (T)	
	SBP	β = −0.41 (p = 4.32 x 10^{-36}) (T)	Giri A. et el. 2018
	Hypertension	β = 0.05 (p = 8.0 x 10^{-16}) (G)	German C. A., 2019
	Hypertension	OR = 0.84 – 0.92 (p = 2.37 x 10^{-8}) (T)	Jeong H. et al., 2020
	SBP	β = −0.027 (p = 3.24 x 10^{-46}) (T)	Sakaue S. et al., 2021
	DBP	β = −0.026 (p = 2.24 x 10^{-48}) (T)	
	PP	β = −0.016 (p = 7.72 x 10^{-18}) (T)	
	MAP	β = −0.028 (p = 3.64 x 10^{-48}) (T)	

Notes: SBP - systolic blood pressure; DBP - diastolic blood pressure; MAP - mean arterial pressure; PP - pulse blood pressure; z-score - standard scores, β – effect, OR – odds ratio, p – significance level.

References

1. Ehret GB, Ferreira T, Chasman DI, et al. The genetics of blood pressure regulation and its target organs from association studies in 342,415 individuals. Nat Genet. 2016;48(10):1171-1184. doi:10.1038/ng.3667
2. Feitosa MF, Kraja AT, Chasman DI, et al. Novel genetic associations for blood pressure identified via gene-alcohol interaction in up to 570K individuals across multiple ancestries. *PLoS One*. 2018;13(6):e0198166. doi:10.1371/journal.pone.0198166
3. German CA, Sinsheimer JS, Klimentidis YC, Zhou H, Zhou JJ. Ordered multinomial regression for genetic association analysis of ordinal phenotypes at Biobank scale. Genet Epidemiol. 2020;44(3):248-260. doi:10.1002/gepi.22276
4. Giri A, Hellwege JN, Keaton JM, et al. Trans-ethnic association study of blood pressure determinants in over 750,000 individuals. Nat Genet. 2019;51(1):51-62. doi:10.1038/s41588-018-0303-9
5. Hartiala JA, Han Y, Jia Q, et al. Genome-wide analysis identifies novel susceptibility loci for myocardial infarction. Eur Heart J. 2021;42(9):919-933. doi:10.1093/eurheartj/ehaa1040
6. Hoffmann TJ, Ehret GB, Nandakumar P, et al. Genome-wide association analyses using electronic health records identify new loci influencing blood pressure variation. Nat Genet. 2017;49(1):54-64. doi:10.1038/ng.3715
7. International Consortium for Blood Pressure Genome-Wide Association Studies, Ehret GB, Munroe PB, et al. Genetic variants in novel pathways influence blood pressure and cardiovascular disease risk. *Nature*. 2011;478(7367):103-109. doi:10.1038/nature10405
8. Jeong H, Jin HS, Kim SS, Shin D. Identifying Interactions between Dietary Sodium, Potassium, Sodium-Potassium Ratios, and FGF5 rs16998073 Variants and Their Associated Risk for Hypertension in Korean Adults. Nutrients. 2020;12(7):2121. doi:10.3390/nu12072121
9. Kato N, Loh M, Takeuchi F, et al. Trans-ancestry genome-wide association study identifies 12 genetic loci influencing blood pressure and implicates a role for DNA methylation. Nat Genet. 2015;47(11):1282-1293. doi:10.1038/ng.3405
10. Koyama S, Ito K, Terao C, et al. Population-specific and trans-ancestry genome-wide analyses identify distinct and shared genetic risk loci for coronary artery disease. Nat Genet. 2020;52(11):1169-1177. doi:10.1038/s41588-020-0705-3
11. Levy D, Ehret GB, Rice K, et al. Genome-wide association study of blood pressure and hypertension. Nat Genet. 2009;41(6):677-687. doi:10.1038/ng.384

12. Liu C, Kraja AT, Smith JA, et al. Meta-analysis identifies common and rare variants influencing blood pressure and overlapping with metabolic trait loci. Nat Genet. 2016;48(10):1162-1170. doi:10.1038/ng.3660

13. Nelson CP, Goel A, Butterworth AS, et al. Association analyses based on false discovery rate implicate new loci for coronary artery disease. Nat Genet. 2017;49(9):1385-1391. doi:10.1038/ng.3913

14. Nikpay M, Goel A, Won HH, et al. A comprehensive 1,000 Genomes-based genome-wide association meta-analysis of coronary artery disease. Nat Genet. 2015;47(10):1121-1130. doi:10.1038/ng.3396

15. Sakaue S, Kanai M, Tanigawa Y, et al. A cross-population atlas of genetic associations for 220 human phenotypes. Nat Genet. 2021;53(10):1415-1424. doi:10.1038/s41588-021-00931-x

16. Shungin D, Winkler TW, Croteau-Chonka DC, et al. New genetic loci link adipose and insulin biology to body fat distribution. Nature. 2015;518(7538):187-196. doi:10.1038/nature14132

17. Sung YJ, Winkler TW, de Las Fuentes L, et al. A Large-Scale Multi-ancestry Genome-wide Study Accounting for Smoking Behavior Identifies Multiple Significant Loci for Blood Pressure. Am J Hum Genet. 2018;102(3):375-400. doi:10.1016/j.ajhg.2018.01.015

18. Surendran P, Drenos F, Young R, et al. Trans-ancestry meta-analyses identify rare and common variants associated with blood pressure and hypertension. Nat Genet. 2016;48(10):1151-1161. doi:10.1038/ng.3654

19. Tachmazidou I, Süveges D, Min JL, et al. Whole-Genome Sequencing Coupled to Imputation Discovers Genetic Signals for Anthropometric Traits. Am J Hum Genet. 2017;100(6):865-884. doi:10.1016/j.ajhg.2017.04.014

20. Takeuchi F, Akiyama M, Matoba N, et al. Interethnic analyses of blood pressure loci in populations of East Asian and European descent. Nat Commun. 2018;9(1):5052. doi:10.1038/s41467-018-07345-0

21. Wain LV, Vaez A, Jansen R, et al. Novel Blood Pressure Locus and Gene Discovery Using Genome-Wide Association Study and Expression Data Sets From Blood and the Kidney. Hypertension. 2017; HYPERTENSIONAHA.117.09438. doi:10.1161/HYPERTENSIONAHA.117.09438

22. Wain LV, Verwoert GC, O'Reilly PF, et al. Genome-wide association study identifies six new loci influencing pulse pressure and mean arterial pressure. Nat Genet. 2011;43(10):1005-1011. doi:10.1038/ng.922

23. Zhou W, Nielsen JB, Fritsche LG, et al. Efficiently controlling for case-control imbalance and sample relatedness in large-scale genetic association studies. Nat Genet. 2018;50(9):1335-1341. doi:10.1038/s41588-018-0184-y

24. Zhu Z, Wang X, Li X, et al. Genetic overlap of chronic obstructive pulmonary disease and cardiovascular disease-related traits: a large-scale genome-wide cross-trait analysis. Respir Res. 2019;20(1):64. doi:10.1186/s12931-019-1036-8
