# Supplementary material for: Effects of Pre-Pregnancy Overweight/Obesity on the Pattern of Association of Hypertension Susceptibility Genes with Preeclampsia
Source: Life (Basel). 2022 Dec 3;12(12):2018. doi: 10.3390/life12122018 (PMC9784908; doi:10.3390/life12122018)
Supplement: Supplementary file 1 [file life-12-02018-s001.zip › Suppl table S3.pdf]

Supplementary table S3

The allele and genotype frequencies of the studied SNPs in the preeclampsia and control groups with preBMI<25

Chr	SNP	Gene	Minor allele	Major allele	Minor allele frequency	Number of the studied chromosomes	Genotype distribution*	H_o	H_e	P_{HWE}
Preeclampsia patients (n=290)										
5	rs1173771	*AC026703.1*	A	G	0.413	566	46/142/95	0.502	0.485	0.624
6	rs1799945	*HFE*	G	C	0.201	572	12/91/183	0.318	0.321	0.854
6	rs805303	*BAG6*	A	G	0.365	580	35/142/113	0.489	0.464	0.377
10	rs932764	*PLCE1*	A	G	0.476	578	65/145/79	0.502	0.499	1.000
10	rs4387287	*OBFC1*	A	C	0.183	542	9/81/181	0.299	0.299	1.000
11	rs633185	*ARHGAP42*	G	C	0.292	568	17/132/135	0.465	0.414	0.045
12	rs7302981	*CERS5*	A	G	0.389	578	43/139/107	0.481	0.475	0.908
12	rs2681472	*ATP2B1*	G	A	0.150	578	7/73/209	0.253	0.256	0.818
17	rs8068318	*TBX2*	C	T	0.299	562	29/110/142	0.391	0.419	0.258
19	rs167479	*RGL3*	G	T	0.460	572	62/139/85	0.486	0.497	0.722
Control group (n=339)										
5	rs1173771	*AC026703.1*	A	G	0.459	652	65/169/92	0.518	0.497	0.503
6	rs1799945	*HFE*	G	C	0.206	676	8/123/207	0.364	0.327	0.045
6	rs805303	*BAG6*	A	G	0.401	676	50/171/117	0.506	0.480	0.366
10	rs932764	*PLCE1*	A	G	0.488	658	85/151/93	0.459	0.499	0.151
10	rs4387287	*OBFC1*	A	C	0.199	632	11/104/201	0.329	0.319	0.724
11	rs633185	*ARHGAP42*	G	C	0.289	672	23/148/165	0.440	0.411	0.231
12	rs7302981	*CERS5*	A	G	0.376	664	40/170/122	0.512	0.469	0.128
12	rs2681472	*ATP2B1*	G	A	0.132	672	4/81/251	0.241	0.229	0.480
17	rs8068318	*TBX2*	C	T	0.265	644	20/131/171	0.407	0.390	0.479
19	rs167479	*RGL3*	G	T	0.520	660	89/165/76	0.500	0.499	1.000

Note: * minor allele homozygotes / heterozygotes / major allele homozygotes
