# Supplementary material for: Effects of Pre-Pregnancy Overweight/Obesity on the Pattern of Association of Hypertension Susceptibility Genes with Preeclampsia
Source: Life (Basel). 2022 Dec 3;12(12):2018. doi: 10.3390/life12122018 (PMC9784908; doi:10.3390/life12122018)
Supplement: Supplementary file 1 [file life-12-02018-s001.zip › Suppl table S4.pdf]

Supplementary table S4

The allele and genotype frequencies of the studied SNPs in the preeclampsia and control groups with preBMI≥25

Chr	SNP	Gene	Minor allele	Major allele	Minor allele frequency	Number of the studied chromosomes	Genotype distribution*	H_o	H_e	P_{HWE}
Preeclampsia patients (n=162)										
5	rs1173771	*AC026703.1*	A	G	0.437	316	29/80/49	0.506	0.492	0.749
6	rs1799945	*HFE*	G	C	0.205	322	11/44/106	0.273	0.326	0.050
6	rs805303	*BAG6*	A	G	0.294	320	12/70/78	0.437	0.415	0.571
10	rs932764	*PLCE1*	A	G	0.462	320	32/84/44	0.525	0.497	0.528
10	rs4387287	*OBFC1*	A	C	0.163	306	5/40/108	0.261	0.273	0.556
11	rs633185	*ARHGAP42*	G	C	0.256	316	10/61/87	0.386	0.381	1.000
12	rs7302981	*CERS5*	A	G	0.332	316	14/77/67	0.487	0.444	0.282
12	rs2681472	*ATP2B1*	G	A	0.152	322	6/37/118	0.230	0.258	0.213
17	rs8068318	*TBX2*	C	T	0.258	314	12/57/88	0.363	0.383	0.533
19	rs167479	*RGL3*	G	T	0.522	318	38/90/31	0.566	0.499	0.113
Control group (n=159)										
5	rs1173771	*AC026703.1*	A	G	0.443	314	28/83/46	0.529	0.493	0.421
6	rs1799945	*HFE*	G	C	0.183	316	4/50/104	0.316	0.300	0.603
6	rs805303	*BAG6*	A	G	0.386	316	29/64/65	0.405	0.474	0.067
10	rs932764	*PLCE1*	A	G	0.459	316	37/71/50	0.449	0.497	0.262
10	rs4387287	*OBFC1*	A	C	0.180	306	3/49/101	0.320	0.295	0.413
11	rs633185	*ARHGAP42*	G	C	0.280	318	12/65/82	0.409	0.403	1.000
12	rs7302981	*CERS5*	A	G	0.383	316	22/77/59	0.487	0.473	0.739
12	rs2681472	*ATP2B1*	G	A	0.154	312	5/38/113	0.244	0.260	0.367
17	rs8068318	*TBX2*	C	T	0.287	314	14/62/81	0.395	0.409	0.697
19	rs167479	*RGL3*	G	T	0.449	316	33/76/49	0.481	0.495	0.748

Note: * minor allele homozygotes / heterozygotes / major allele homozygotes
